# CD25 and CD69 induction by α4β1 outside-in signalling requires TCR early signalling complex proteins

**DOI:** 10.1042/BJ20130485

**Published:** 2013-07-26

**Authors:** Ann-Marie Cimo, Zamal Ahmed, Bradley W. McIntyre, Dorothy E. Lewis, John E. Ladbury

**Affiliations:** *Department of Biochemistry and Molecular Biology, The University of Texas MD Anderson Cancer Center, Houston, TX 77030, U.S.A.; †Department of Immunology, The University of Texas MD Anderson Cancer Center, Houston, TX 77030, U.S.A.; ‡Department of Internal Medicine – Infectious Diseases, The University of Texas Health Science Center at Houston Medical School, Houston, TX 77030, U.S.A.

**Keywords:** early signalling complex, extracellular-signal-regulated kinase (ERK), integrin, outside-in signalling, T-cell co-stimulation, CXCR4, CXC chemokine receptor 4, ECM, extracellular matrix, ERK, extracellular-signal-regulated kinase, ESC, early signalling complex, FTI, farnesyl transferase inhibitor, GDS, guanine nucleotide dissociation stimulator, HRP, horseradish peroxidase, IFNαR, interferon-α receptor, LAT, linker for activation of T-cells, Lck, lymphocyte-specific kinase, LFA-1, lymphocyte-function-associated antigen 1, MAPK, mitogen-activated protein kinase, MEK, MAPK/ERK kinase, PBMC, peripheral blood mononuclear cell, RBD, Rap1-binding domain, SH2, Src homology 2, SLP-76, SH2-domain-containing leukocyte protein of 76 kDa, TCR, T-cell receptor, VCAM-1, vascular cell adhesion molecule-1, VLA-4, very late antigen-4, ZAP-70, ζ-chain-associated protein of 70 kDa

## Abstract

Distinct signalling pathways producing diverse cellular outcomes can utilize similar subsets of proteins. For example, proteins from the TCR (T-cell receptor) ESC (early signalling complex) are also involved in interferon-α receptor signalling. Defining the mechanism for how these proteins function within a given pathway is important in understanding the integration and communication of signalling networks with one another. We investigated the contributions of the TCR ESC proteins Lck (lymphocyte-specific kinase), ZAP-70 (ζ-chain-associated protein of 70 kDa), Vav1, SLP-76 [SH2 (Src homology 2)-domain-containing leukocyte protein of 76 kDa] and LAT (linker for activation of T-cells) to integrin outside-in signalling in human T-cells. Lck, ZAP-70, SLP-76, Vav1 and LAT were activated by α4β1 outside-in signalling, but in a manner different from TCR signalling. TCR stimulation recruits ESC proteins to activate the mitogen-activated protein kinase ERK (extracellular-signal-regulated kinase). α4β1 outside-in-mediated ERK activation did not require TCR ESC proteins. However, α4β1 outside-in signalling induced CD25 and co-stimulated CD69 and this was dependent on TCR ESC proteins. TCR and α4β1 outside-in signalling are integrated through the common use of TCR ESC proteins; however, these proteins display functionally distinct roles in these pathways. These novel insights into the cross-talk between integrin outside-in and TCR signalling pathways are highly relevant to the development of therapeutic strategies to overcome disease associated with T-cell deregulation.

## INTRODUCTION

Integrins are critical for T-cell function, including T-cell recirculation and recruitment into inflammatory sites, the formation of conjugates with antigen-presenting cells and cytokine secretion. Integrins also play a major role in T-cell activation by providing co-stimulatory signals that synergize with early signals initiated by the TCR (T-cell receptor). The predominant integrins expressed on T-cells include VLA-4 (very late antigen-4; α4β1) and LFA-1 (lymphocyte-function-associated antigen 1; αLβ2). Integrins are capable of signalling bidirectionally in pathways referred to as inside-out and outside-in signalling. Inside-out signalling can be induced by intracellular signals triggered by the engagement of other cell-surface receptors, such as TCR and chemokine receptors. In outside-in signalling, integrins transmit signals from the exterior environment to the interior of the cell upon integrin ligand binding.

The signalling events immediately following TCR stimulation are well characterized and involve the recruitment and assembly of a complex of proteins known as the TCR ESC (early signalling complex). TCR ESC proteins, including Lck (lymphocyte-specific kinase), ZAP-70 (ζ-chain-associated protein of 70 kDa), Vav1, LAT (linker for activation of T-cells) and SLP-76 [SH2 (Src homology 2)-domain-containing leukocyte protein of 76 kDa], form a multimolecular signalling complex that ultimately results in the activation of the ERK (extracellular-signal-regulated kinase)/MAPK (mitogen-activated protein kinase) pathway in T-cells. Activation of this pathway is essential to various T-cell processes, including proliferation and differentiation. The TCR ESC was initially thought to be exclusively part of TCR signalling; however, other receptor pathways on T-cells, including IFNαR (interferon-α receptor) and CXCR4 (CXC chemokine receptor 4), are integrated through the common use of this subset of proteins [1–7]. Although TCR ESC proteins have been shown to be involved in integrin inside-out signalling mediated by TCR and chemokines, little is known about the role of these proteins in integrin outside-in signalling [8–10]. Improved understanding of the molecular basis that governs the communication between α4β1 outside-in and TCR signalling networks will be valuable in the design of novel therapeutics that manipulate the immune system to overcome T-cell-based disorders such as cancer and autoimmune disease.

In the present study, we investigated the involvement of TCR ESC proteins in α4β1 outside-in signalling in human T-cells and found that α4β1 outside-in and TCR signalling pathways are networked through the adoption of common TCR ESC machinery. However, the activation of TCR ESC proteins was markedly different between α4β1 outside-in and TCR signalling pathways. In addition, our studies revealed that TCR ESC proteins mediate distinct functions in these signal transduction pathways.

## MATERIALS AND METHODS

### Cells

The human Jurkat (clone E6.1) acute T-cell leukaemia cell line and the TCRβ-deficient JRT3-T3.5, Lck-deficient JCAM1.6, and ZAP-70-deficient P116 Jurkat cell lines were obtained from A.T.C.C. (Manassas, VA, U.S.A.). The Lck-reconstituted JCAM1.6 cell line (JCAM1.6^WT^Lck) was a gift from Dr Gordon Mills (Department of Systems Biology, MD Anderson Cancer Center, Houston, TX, U.S.A.). The SLP-76-deficient J14, SLP-76-reconstituted J14 (J14^WT^SLP-76) and ZAP-70-reconstituted P116 (P116^WT^ZAP-70) cell lines were a gift from Dr Arthur Weiss (Department of Medicine, Hughes Medical Institute, University of California San Francisco, CA, U.S.A.). The Vav1-deficient J.Vav1 cell line and Vav1-reconstituted J.Vav1 (J.Vav1^WT^Vav1) cell lines were a gift from Dr Robert Abraham (Program in Signal Transduction, The Burnham Institute, La Jolla, CA, U.S.A.). The LAT-deficient JCAM2.5 and LAT-reconstituted JCAM2.5 (JCAM2.5^WT^LAT) cell lines were a gift from Dr Lawrence Samelson (Center for Cancer Research, National Cancer Institute, Bethesda, MD, U.S.A.). All cells were cultured in RPMI-1640 medium (Hyclone Laboratories) supplemented with 10% heat-inactivated FBS (Hyclone) and antibiotics/antimycotics (100 units/ml penicillin, 100 μg/ml streptomycin and 1.25 μg/ml Fungizone) (Invitrogen). J.Vav1 cells were cultured in the above medium supplemented with 500 μg/ml G418 (Invitrogen). Cells were maintained at 37°C under 5% CO_2_ in a humidified incubator.

PBMCs (peripheral blood mononuclear cells) were isolated from buffy coats purchased from the Gulf Coast Regional Blood Center (Houston, TX, U.S.A.). PBMCs were isolated through Ficoll density-gradient cell separation on Ficoll-Paque PLUS (GE Healthcare). Naïve CD4^+^ T-cells were isolated from PBMCs by negative selection using the EasySep Human Naïve CD4+ T Cell Enrichment Kit (StemCell Technologies) according to the manufacturer's instructions. The purity of the resultant T-cell population was routinely 94% as determined by flow cytometric analysis.

### Integrin outside-in and TCR activation

α4β1 outside-in integrin signalling was induced by using the highly specific monoclonal anti-α4β1 antibody 19H8 followed by the addition of a secondary cross-linker. 19H8 was made in the laboratory of Dr Bradley McIntyre (Department of Immunology, The University of Texas MD Anderson Cancer Center) [11]. Serum-starved TCR ESC knockout and reconstituted Jurkat cell lines were treated with 19H8 (1 μg/ml) and incubated on ice for 40 min. Cells were washed twice in cold serum-free RPMI-1640 medium and treated with the secondary cross-linker (AffiniPure F[ab’]2 rabbit anti-mouse IgG; Jackson ImmunoResearch Laboratories) at time points ranging from 5 to 30 min. Fibronectin, an α4β1 ligand, was also used to induce α4β1 outside-in signalling. Serum-starved Jurkat cells were added to fibronectin-coated plates (BD Biocoat; BD Biosciences) at time points ranging from 5 to 30 min. The non-integrin ligand poly-L-lysine was used as a negative control. Tissue culture plates containing 24 wells (Corning) were precoated with poly-L-lysine (0.1 mg/ml; R&D Systems) for 2 h at room temperature (25°C) and washed three times with serum-free RPMI-1640 medium. For TCR stimulation, 24-well tissue culture-treated plates were precoated with the anti-CD3 antibody UCHT1 (1 μg/ml; R&D Systems) in PBS overnight at 4°C. Plates were blocked with 2% (w/v) BSA in PBS for 2 h at room temperature and washed three times with serum-free RPMI-1640 medium. Serum-starved TCR ESC knockout and reconstituted Jurkat cell lines were added to the UCHT1-coated plates at time points ranging from 5 to 30 min. Whole-cell lysates were obtained for Western blot analysis as described previously [7].

### Integrin co-stimulation

Tissue culture-treated plates containing 24 wells were precoated with the anti-CD3 antibody UCHT1 as described above. TCR ESC knockout and reconstituted Jurkat cell lines were stimulated with soluble 19H8 using cross-linking and added to the UCHT1-coated plates for 30 min. Naïve CD4^+^ T-cells were co-stimulated with UCHT1 and 19H8 in a similar manner as the Jurkat cell lines; however, they were stimulated for 24 h. In addition, Lck inhibitor II and piceatannol (EMD Biosciences) were used to inhibit Lck and ZAP-70 in primary CD4^+^ T-cells respectively. Cells were pre-treated with Lck inhibitor II (20 nM) or piceatannol (50 μg/ml) for 2 h prior to 19H8 cross-linking. CD25 and CD69 expression in treated cells were determined by Western blotting.

### Ras and Rap1 activation

Lysates were obtained from Jurkat cells that were either untreated or treated with 19H8 plus cross-linker for 5, 10 and 30 min. Ras-GTP and Rap1-GTP levels in the lysates were assayed using specific Ras (Cytoskeleton) and Rap1 (Pierce) Activation kits respectively, according to the manufacturers’ instructions. A GST-tagged Ras-binding domain of Raf-1 fusion protein conjugated to glutathione agarose beads was used to affinity precipitate active Ras (Ras-GTP) from the cell lysates. A GST-fusion protein containing the RBD (Rap1-binding domain) of human Ra1GDS (guanine nucleotide dissociation stimulator) was used to affinity precipitate active Rap1 (Rap1-GTP) from the cell lysates. The GST–Ra1GDS–RBD fusion protein was incubated with the cell lysate and a glutathione resin. The amount of Ras-GTP and Rap1-GTP in each pulldown precipitate and total Ras and Rap1 expression in the cell lysates was analysed by Western blotting.

### Ras and ERK inhibition

The FTIs (farnesyl transferase inhibitors) manumycin A (EMD Biosciences) and B581 (Cayman Chemical) were used to inhibit Ras. Jurkat cells were pre-treated with manumycin A (10 μM) or B581 (50 μM) for 30 min prior to integrin cross-linking. Jurkat cells were also pre-treated with B581 (50 μM) for 30 min prior to UCHT1 stimulation. The MEK (MAPK/ERK kinase) inhibitor U0126 (Cell Signaling) was used to inhibit ERK. Jurkat cells were pre-treated with U0126 (10 μM) for 2 h prior to integrin cross-linking. Treated cells were then analysed by Western blotting.

### B-Raf and Rap1 siRNA

Transfection of Accell non-targeting and B-Raf-specific siRNAs (Dharmacon) and Rap1-specific and non-targeting siRNAs (Santa Cruz Biotechnology) in Jurkat cells was performed according to the manufacturers’ instructions. Transfected cells were used in an integrin cross-linking assay and analysed by Western blotting.

### Western blot

Western blot analysis was performed on whole-cell lysates as described previously [7]. The antibodies against the following were used for Western blotting: phospho-(Thr^202^/Tyr^204^)-ERK1/2, ERK1/2, phospho-Tyr^191^-LAT, LAT, Lck, phospho-(Ser^217^/Ser^221^)-MEK1/2, MEK1/2, phospho-Ser^338^-Raf-1, Raf-1, Rap1, SLP-76, phospho-Tyr^319^-ZAP-70, phospho-Tyr^493^-ZAP-70, ZAP-70, B-Raf, CD25 and HRP (horseradish peroxidase)-conjugated anti-mouse secondary antibody (all from Cell Signaling); phospho-Tyr^113^-SLP-76 and phospho-Tyr^174^-Vav1 (Abcam); Vav1, phospho-Tyr^394^-Lck, phospho-(Thr^598^/Ser^601^)-B-Raf, CD69 and HRP-conjugated goat anti-rabbit IgG secondary antibody (all from Santa Cruz Biotechnology); phospho-Tyr^128^-SLP-76 polyclonal antibody (Assay Biotechnology); and β-actin monoclonal antibody (Sigma–Aldrich).

## RESULTS

### ZAP-70 phosphorylation at Tyr^319^ is Lck-dependent in α4β1 outside-in and TCR signalling pathways

**Figure 1:**
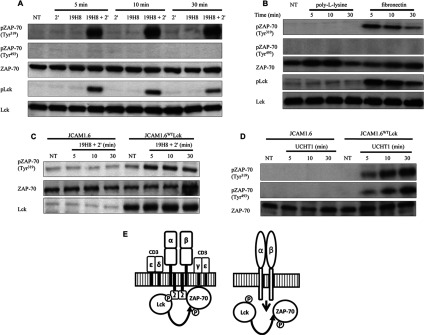
ZAP-70 phosphorylation at Tyr^319^ is Lck dependent in α4β1 outside-in and TCR signalling pathways (**A**) Jurkat cells were stimulated with the anti-α4β1 antibody 19H8 followed by the addition of a secondary cross-linker (2′) for the indicated time points. Expression of phospho-Tyr^394^-Lck, phospho-Tyr^319^-ZAP-70, phospho-Tyr^493^-ZAP-70, Lck and ZAP-70 was determined by Western blot analysis. Non-stimulated cells (NT) and cells treated with either 2′ or 19H8 alone were used as controls. (**B**) Jurkat cells were added to 24-well fibronectin-coated plates for the indicated time points. Poly-L-lysine was used as a negative control. Expression of phospho-Tyr^394^-Lck, phospho-Tyr^319^-ZAP-70, phospho-Tyr^493^-ZAP-70, Lck and ZAP-70 was determined by Western blot analysis. (**C**) Lck-deficient JCAM1.6 and Lck reconstituted JCAM1.6 (JCAM1.6^WT^Lck) Jurkat cells were stimulated with 19H8 followed by the addition of 2′ for the indicated time points. Expression of phospho-Tyr^319^-ZAP-70, ZAP-70 and Lck was determined by Western blot analysis. (**D**) JCAM1.6 and JCAM1.6^WT^Lck cells were stimulated with the anti-CD3 antibody UCHT1 for the indicated time points. Expression of phospho-Tyr^319^-ZAP-70, phospho-Tyr^493^-ZAP-70 and ZAP-70 was determined by Western blot analysis. (**E**) Schematic representation of Lck and ZAP-70 activation following stimulation of TCR (left-hand panel) and α4β1 outside-in (right-hand panel) signalling pathways. (P), phosphorylation; NT, not treated.

The effect of integrin outside-in signalling on the activation of TCR ESC proteins in Jurkat cells was determined using the specific anti-α4β1 integrin antibody 19H8 followed by the addition of a secondary cross-linker (2′). Lck and ZAP-70 are protein tyrosine kinases that are initially activated following TCR stimulation. TCR signalling induces Lck kinase activity through autophosphorylation at Tyr^394^. Activated Lck recruits ZAP-70 to the TCR, resulting in ZAP-70 autophosphorylation at Tyr^319^ and binding to Lck with subsequent phosphorylation of ZAP-70 at Tyr^493^ [12,13]. Phosphorylation of ZAP-70 at Tyr^319^ is crucial for ZAP-70 activation, the phosphorylation of the ZAP-70 substrates SLP-76 and LAT, and Ras activation [13,14]. Phosphorylation at Tyr^493^ in the activation loop is required for ZAP-70 catalytic activation. In contrast with untreated cells and cells treated with either 19H8 alone or cross-linker alone, Lck activity was induced by the cross-linking of 19H8 and remained activated throughout the 30 min time course (Figure 1A). 19H8 cross-linking resulted in the phosphorylation of ZAP-70 at Tyr^319^, but not Tyr^493^, throughout the 30 min time period (Figure 1A). An α4β1 ligand, fibronectin, was also used to confirm the activation of ZAP-70 and Lck by α4β1 outside-in signalling. Phosphorylation of Lck and ZAP-70 (Tyr^319^) was induced by fibronectin but not by non-specific adhesion to poly-L-lysine (Figure 1B). Thus α4β1 outside-in signalling results in the autophosphorylation of ZAP-70, but not its full catalytic activation. To determine whether Lck recruits ZAP-70 in α4β1 outside-in signalling, ZAP-70 phosphorylation at Tyr^319^ was determined in JCAM1.6 and JCAM1.6^WT^Lck cells upon 19H8 cross-linking. Lck expression was absent in JCAM1.6 cells, but was present in JCAM1.6^WT^Lck cells (Figure 1C). As in TCR signalling, ZAP-70 phosphorylation at Tyr^319^ was induced by α4β1 outside-in signalling in JCAM1.6^WT^Lck, but not JCAM1.6, cells (Figures 1C and 1D). Thus α4β1 outside-in and TCR signalling pathways result in the activation of Lck, which is required for the recruitment and autophosphorylation of ZAP-70 (Figure 1E). However, in contrast with α4β1 outside-in signalling, TCR stimulation induced Lck-dependent ZAP-70 phosphorylation at Tyr^493^ (Figure 1D). Together, these data indicate that α4β1 outside-in signalling induces only Lck-dependent ZAP-70 phosphorylation on Tyr^319^ and not catalytic function. TCR signalling, on the other hand, requires Lck to stimulate both the phosphorylation and kinase activity of ZAP-70.

### Activation of Vav1 and LAT is ZAP-70 independent in α4β1 outside-in signalling

**Figure 2:**
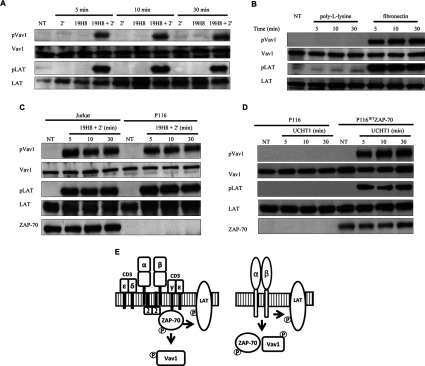
Activation of Vav1 and LAT by α4β1 outside-in signalling is ZAP-70 independent (**A**) Jurkat cells were stimulated with the anti-α4β1 antibody 19H8 followed by the addition of a secondary cross-linker (2′) for the indicated time points. Expression of phospho-Tyr^174^-Vav1, phospho-Tyr^191^-LAT, Vav1 and LAT was determined by Western blot analysis. (**B**) Jurkat cells were added to 24-well fibronectin-coated plates for the indicated time points. Poly-L-lysine was used as a negative control. Expression of phospho-Tyr^174^-Vav1, phospho-Tyr^191^-LAT, Vav1 and LAT was determined by Western blot analysis. (**C**) Parental and ZAP-70-deficient P116 Jurkat cells were stimulated with 19H8 followed by the addition of 2′ for the indicated time points. Expression of phospho-Tyr^174^-Vav1, phospho-Tyr^191^-LAT, Vav1, LAT and ZAP-70 was determined by Western blot analysis. (**D**) ZAP-70-deficient P116 and ZAP-70-reconstituted P116 (P116^WT^ZAP-70) Jurkat cells were stimulated with the anti-CD3 antibody UCHT1 for the indicated time points. Expression of phospho-Tyr^174^-Vav1, phospho-Tyr^191^-LAT, Vav1, LAT, and ZAP-70 was determined by Western blot analysis. (**E**) Schematic representation of Vav1 and LAT activation following stimulation of TCR (left-hand panel) and α4β1 outside-in (right-hand panel) signalling pathways. (P), phosphorylation; NT, not treated.

In TCR signalling, phosphorylation of the adaptor proteins Vav1 (Tyr^174^), SLP-76 (Tyr^113^, Tyr^128^ and Tyr^145^) and LAT (Tyr^132^, Tyr^171^, Tyr^191^ and Tyr^226^) by ZAP-70 is critical to their function [15–17]. To determine whether Vav1 and LAT were activated by α4β1 outside-in signalling, we examined the site-specific phosphorylation kinetics of Tyr^174^ on Vav1 and one site on LAT, Tyr^191^. Vav1 and LAT were phosphorylated throughout the 30 min time course in response to 19H8 cross-linking and fibronectin (Figures 2A and 2B). To ascertain whether the α4β1-mediated activation of Vav1 and LAT was dependent on ZAP-70, Vav1 and LAT phosphorylation were determined in ZAP-70-deficient P116 Jurkat cells. Vav1 and LAT phosphorylation induced by 19H8 cross-linking were similar between Jurkat and P116 cells (Figure 2C), whereas Vav1 and LAT phosphorylation were absent in TCR-stimulated P116 cells, but were present in ZAP-70-reconstituted P116 cells (P116^WT^ZAP-70) (Figure 2D). In contrast with TCR signalling, Vav1 and LAT activation were independent of ZAP-70 in α4β1 outside-in signalling (Figure 2E). This is consistent with the observations above (Figure 1), where ZAP-70 undergoes Lck-dependent tyrosine phosphorylation without ZAP-70 becoming an active kinase. In this context, ZAP-70 appears to act as a scaffold protein in integrin signalling, whereas it functions as a scaffold and kinase in TCR signalling [18–20].

### Vav1 phosphorylation is independent of SLP-76 in α4β1 outside-in signalling

**Figure 3:**
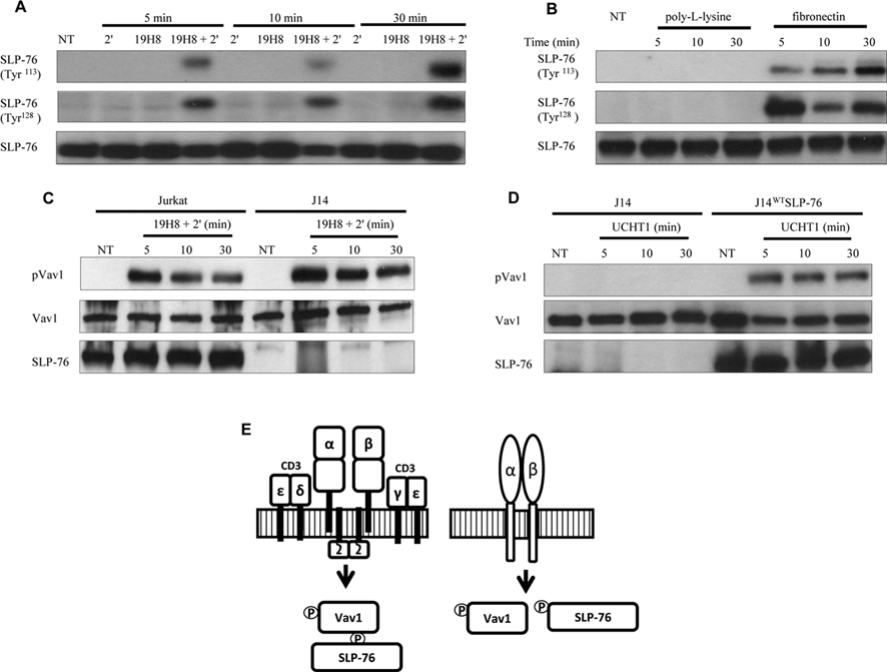
SLP-76 is not required for Vav1 phosphorylation in α4β1 outside-in signalling (**A**) Jurkat cells were stimulated with the anti-α4β1 antibody 19H8 followed by the addition of a secondary cross-linker (2′) for the indicated time points. Expression of phospho-Tyr^113^-SLP-76, phospho-Tyr^128^-SLP-76 and SLP-76 was determined by Western blot analysis. (**B**) Jurkat cells were added to 24-well fibronectin-coated plates for the indicated time periods. Poly-L-lysine was used as a negative control. Expression of phospho-Tyr^113^-SLP-76, phospho-Tyr^128^-SLP-76 and SLP-76 was determined by Western blot analysis. (**C**) Parental and SLP-76-deficient J14 Jurkat cells were stimulated with 19H8 followed by the addition of 2′ for the indicated time points. Expression of phospho-Tyr^174^-Vav1, Vav1 and SLP-76 was determined by Western blot analysis. (**D**) J14 and SLP-76-reconstituted J14 (J14^WT^SLP-76) Jurkat cells were stimulated with the anti-CD3 antibody UCHT1 for the indicated time points. Expression of phospho-Tyr^174^-Vav1, Vav1 and SLP-76 was determined by Western blot analysis. (**E**) Schematic representation of Vav1 recruitment by SLP-76 following stimulation of TCR (left-hand panel) and α4β1 outside-in (right-hand panel) signalling pathways. (P), phosphorylation; NT, not treated.

ZAP-70 phosphorylates SLP-76 at specific sites (Tyr^113^ and Tyr^128^) that allow Vav1 SH2 domain binding and facilitate Vav1 recruitment to the TCR ESC for phosphorylation [15,21]. Therefore we investigated whether α4β1 outside-in signalling induced SLP-76 phosphorylation and its role in Vav1 phosphorylation. SLP-76 was phosphorylated at Tyr^113^ and Tyr^128^ throughout the 30 min time course in response to 19H8 cross-linking and fibronectin (Figures 3A and 3B). Despite the phosphorylation of Vav1-binding sites on SLP-76, Vav1 was phosphorylated in response to 19H8 cross-linking in both parental and SLP-76-deficient J14 Jurkat cells (Figure 3C). In contrast, Vav1 phosphorylation was dependent on SLP-76 expression in TCR-stimulated cells, as indicated by the lack of Vav1 phosphorylation in J14 cells and its restoration in J14^WT^SLP-76 cells (Figure 3D). Therefore SLP-76 is activated in response to integrin α4β1 outside-in signalling, but it is not involved in Vav1 phosphorylation (Figure 3E). Perhaps this is not surprising, because the TCR-stimulated SLP-76–Vav1 interaction is dependent on ZAP-70 kinase function, which is not active in integrin signalling. Taken together, these results indicate that α4β1 outside-in signalling leads to the phosphorylation of the TCR ESC proteins Lck, ZAP-70, Vav1, SLP-76 and LAT and therefore this pathway utilizes a subset of proteins common to TCR signalling. However, the activation of TCR ESC proteins in response to TCR stimulation is not replicated in α4β1 outside-in signalling.

### α4β1 outside-in signalling activates ERK via a Rap1/B-Raf/MEK pathway

**Figure 4:**
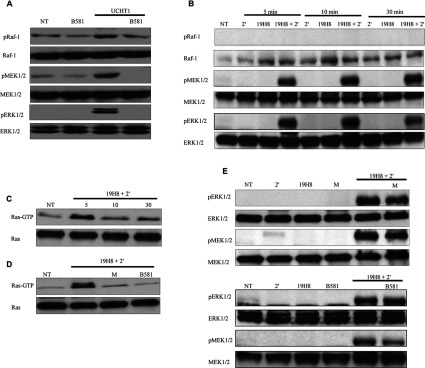
α4β1 outside-in signalling activates ERK independently of Ras and Raf-1 (**A**) Jurkat cells were stimulated with the anti-CD3 antibody UCHT1 for 5 min. Jurkat cells were also pre-treated with the FTI B581 (50 μM) for 30 min prior to UCHT1 stimulation for 5 min. Expression of phospho-(Thr^202^/Tyr^204^)-ERK1/2, phospho-Ser^338^-Raf-1, phospho-(Ser^217^/Ser^221^)-MEK1/2, ERK1/2, Raf-1 and MEK1/2 was determined by Western blot analysis. Non-stimulated cells (NT) and cells treated with B581 alone were used as controls. (**B**) Jurkat cells were stimulated with the anti-α4β1 antibody 19H8 followed by the addition of a secondary cross-linker (2′) for the indicated time points. Expression of phospho-(Thr^202^/Tyr^204^)-ERK1/2, phospho-Ser^338^-Raf-1, phospho-(Ser^217^/Ser^221^)-MEK1/2, ERK1/2, Raf-1 and MEK1/2 was determined by Western blot analysis. NT (not treated) cells and cells treated with either 2′ or 19H8 alone were used as controls. (**C**) Jurkat cells were stimulated with 19H8 followed by the addition of 2′ for the indicated time points. A GST-tagged Ras-binding domain of Raf-1 fusion protein conjugated to glutathione–agarose beads was used to affinity precipitate Ras-GTP from the cell lysates. Ras-GTP and total Ras levels were determined by Western blot analysis. (**D**) FTI-mediated inhibition of Ras activity induced by 19H8 cross-linking was confirmed by determining Ras-GTP levels. (**E**) Jurkat cells were stimulated with 19H8 followed by the addition of 2′ for 5 min. Jurkat cells were also pre-treated with the FTIs manumycin A (M; 10 μM) or B581 (50 μM) for 30 min prior to the addition of 2′ for 5 min. Expression of phospho-(Thr^202^/Tyr^204^)-ERK1/2, phospho-(Ser^217^/Ser^221^)-MEK1/2, ERK1/2 and MEK1/2 was determined by Western blot analysis. NT and cells treated with either 2′, 19H8, M or B581 alone were used as controls.

A major outcome of TCR-mediated assembly of the ESC is activation of the MAPK ERK via the canonical Ras/Raf-1/MEK pathway. As shown in Figure 4(A), inhibition of Ras using the FTI B581 suppressed Raf-1, MEK and ERK activation by the TCR. Integrin outside-in signalling induced by antibody cross-linking and ECM (extracellular matrix) substrates has been shown to activate ERK in Jurkat cells [22–24]. Therefore we confirmed ERK activation by α4β1 outside-in signalling and determined the pathway involved. In contrast with untreated cells and cells treated with either integrin antibody alone or 2′ alone, ERK and MEK, but not Raf-1, phosphorylation were induced by 19H8 cross-linking throughout the 30 min time course (Figure 4B). To determine whether MEK and ERK activation were Ras dependent, we first determined whether Ras was activated. Active Ras (Ras-GTP) levels were increased by 19H8 cross-linking throughout the 30 min time course; however, Ras-GTP was substantially higher at the 5 min time point in comparison with the 10 and 30 min time points (Figure 4C). To determine the involvement of Ras in the ERK response, the FTIs manumycin A and B581 were used to block Ras activity. Manumycin A and B581 effectively inhibited Ras activity induced by 19H8 cross-linking; however, ERK and MEK phosphorylation were unaffected (Figures 4D and 4E). These data indicate that integrin α4β1 outside-in activation of ERK is independent of Ras and Raf-1.

**Figure 5:**
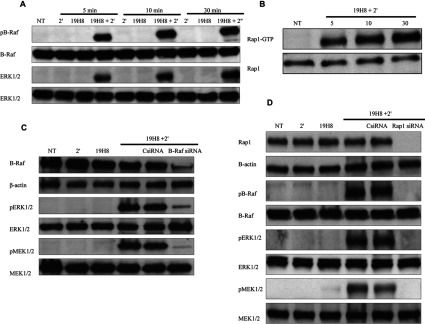
α4β1 outside-in signalling induces ERK activation via a Rap1/B-Raf/MEK pathway (**A**) Jurkat cells were stimulated with the anti-α4β1 antibody 19H8 followed by the addition of a secondary cross-linker (2′) for the indicated time points. Expression of phospho-(Thr^598^/Ser^601^)-B-Raf, phospho-(Thr^202^/Tyr^204^)-ERK1/2, B-Raf and ERK1/2 was determined by Western blot analysis. NT (not-treated) cells and cells treated with either 2′ or 19H8 alone were used as controls. (**B**) Jurkat cells were stimulated with 19H8 followed by the addition of 2′ for the indicated time periods. A GST-fusion protein containing the RBD of human Ra1GDS was used to affinity precipitate Rap1-GTP from the cell lysates. Rap1-GTP and total Rap1 levels were determined by Western blot analysis. (**C**) Jurkat cells transfected with specific B-Raf siRNA or a non-targeting control siRNA (CsiRNA) were stimulated with 19H8 followed by the addition of 2′ for 5 min. Expression of phospho-(Thr^202^/Tyr^204^)-ERK1/2, phospho-(Ser^217^/Ser^221^)-MEK1/2, B-Raf, ERK1/2 and MEK1/2 was determined by Western blotting. To demonstrate that siRNA-mediated knockdown of B-Raf expression was not due to differences in protein loading, β-actin was used as a loading control. (**D**) Jurkat cells transfected with specific Rap1 siRNA or CsiRNA were stimulated with 19H8 followed by the addition of 2′ for 5 min. Expression of phospho-(Thr^202^/Tyr^204^)-ERK1/2, phospho-(Ser^217/221^)-MEK1/2, phospho-(Thr^598^/Ser^601^)-B-Raf, Rap1, ERK1/2, MEK1/2, B-Raf and β-actin was determined by Western blot analysis.

As α4β1 outside-in signalling did not adhere to the canonical Ras/Raf-1/MEK pathway of ERK activation, we investigated the potential involvement of Rap1 and B-Raf. Rap1 and B-Raf have been shown to be potent MEK/ERK activators [25–28]. B-Raf phosphorylation and Rap1 activation were induced throughout the 30 min time course in response to 19H8 cross-linking (Figures 5A and 5B). An siRNA-based approach was used to determine the involvement of Rap1 and B-Raf in α4β1-mediated ERK activation. B-Raf and Rap1 siRNA were effective in the knockdown of B-Raf and Rap1 expression respectively, whereas the control non-targeting siRNAs (CsiRNAs) had no effect on the expression of these proteins (Figures 5C and 5D). Knockdown of either B-Raf or Rap1 expression inhibited MEK and ERK activation induced by 19H8 cross-linking (Figures 5C and 5D). Furthermore, inhibition of Rap1 expression blocked the activation of B-Raf (Figure 5D). Therefore α4β1 outside-in signalling uses Rap1/B-Raf/MEK to induce ERK activation.

### A functional TCR and TCR ESC are not required for ERK activation induced by α4β1 outside-in signalling

**Figure 6:**
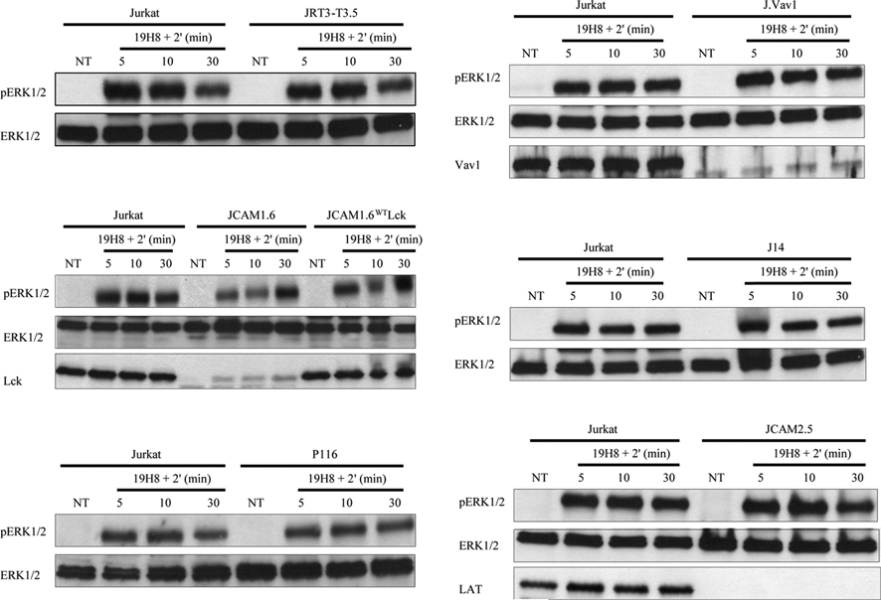
A functional TCR and TCR ESC proteins are not required for ERK activation induced by α4β1 outside-in signalling Parental, TCRβ-deficient JRT3-T3.5, Lck-deficient JCAM1.6, Lck-reconstituted JCAM1.6 (JCAM1.6^WT^Lck), ZAP-70-deficient P116, Vav1-deficient J.Vav1, SLP-76-deficient J14 and LAT-deficient JCAM2.5 Jurkat cells were stimulated with the anti-α4β1 antibody 19H8 followed by the addition of a secondary cross-linker (2′) for the indicated time points. Expression of phospho-(Thr^202^/Tyr^204^)-ERK1/2 and ERK1/2 was determined by Western blot analysis. NT, not treated.

Both IFNαR and CXCR4 require the TCR and associated ESC members to activate ERK in T-cells [1–7]. To determine whether ERK activation induced by α4β1 outside-in signalling requires an intact TCR and associated ESC members, TCRβ-deficient JRT3-T3.5 and TCR ESC knockout Jurkat cell lines were used. We have previously demonstrated that JRT3-T3.5 cells failed to induce ERK phosphorylation in response to TCR stimulation in Jurkat cells [7]. Stimulation of outside-in signalling by 19H8 cross-linking induced comparable ERK phosphorylation in parental and JRT3-T3.5, P116, J.Vav1, J14 and JCAM2.5 Jurkat cells (Figure 6). ERK activation was diminished in JCAM1.6 cells compared with parental cells at the earlier 5 and 10 min time points, whereas the ERK response at 30 min was similar between these cell lines (Figure 6). ERK activation profiles were comparable between parental cells and Lck-reconstituted JCAM1.6 cells (JCAM1.6^WT^Lck) (Figure 6). These data indicate that α4β1 outside-in signalling activates ERK via a mechanism independent of the TCR and TCR ESC proteins ZAP-70, Vav1, SLP-76 and LAT. However, the results of the present study do suggest that Lck is involved in α4β1-mediated ERK activation.

### CD25 expression induced by α4β1 outside-in signalling requires TCR ESC proteins

**Figure 7:**
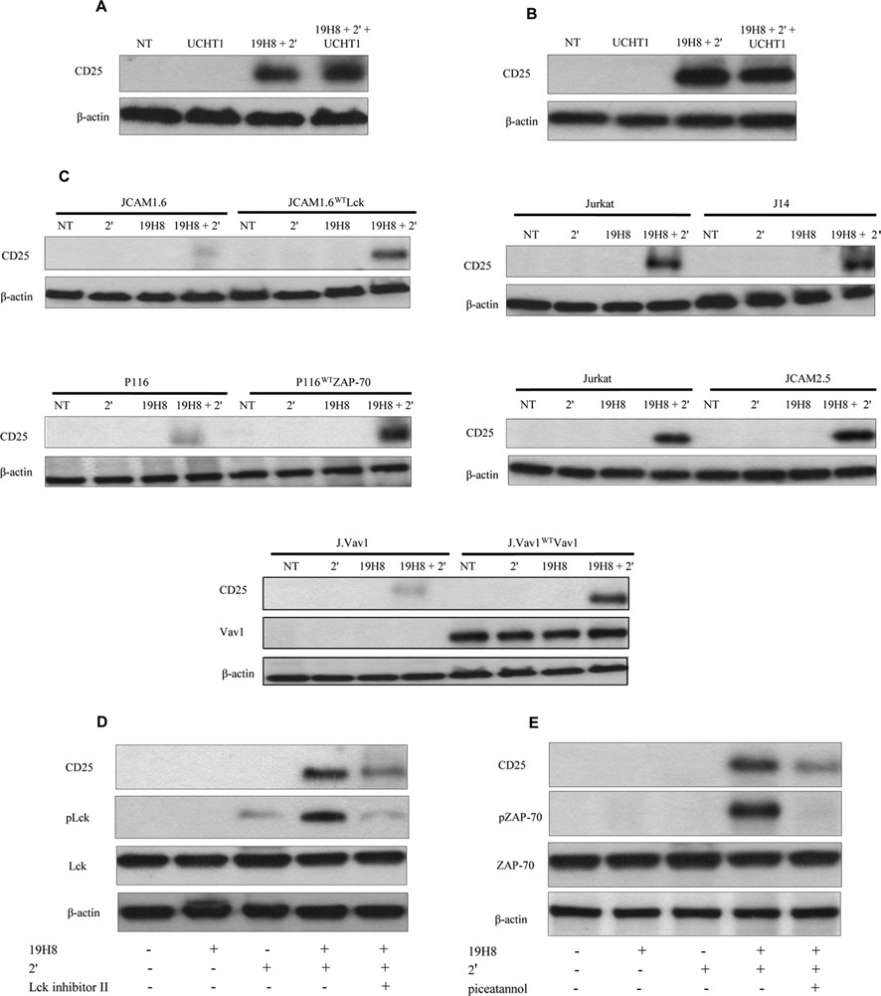
Induction of CD25 expression by α4β1 outside-in signalling requires TCR ESC proteins (**A**) Jurkat cells and (**B**) naïve CD4^+^ T-cells were stimulated with the anti-CD3 antibody UCHT1, the anti-α4β1 antibody 19H8 plus secondary cross-linker (2′) or UCHT1 in combination with 19H8 plus 2′ for 30 min. CD25 expression was determined by Western blot analysis. β-actin was used as a loading control. (**C**) Parental, Lck-deficient JCAM1.6, Lck-reconstituted JCAM1.6 (JCAM1.6^WT^Lck), ZAP-70-deficient P116, ZAP-70-reconstituted P116 (P116^WT^ZAP-70), Vav1-deficient J.Vav1, Vav1-reconstituted J.Vav1 (J.Vav1^WT^Vav1), SLP-76-deficient J14 and LAT-deficient JCAM2.5 Jurkat cells were stimulated with 19H8 plus 2′ for 30 min. Expression of CD25 expression was determined by Western blotting. β-actin was used as a loading control. (**D**) Naïve CD4^+^ T-cells were pre-treated for 2 h with Lck inhibitor II (20 nM) or the (**E**) ZAP-70 inhibitor piceatannol (50 μg/ml) prior to stimulation with 19H8 plus 2′ for 24 h. Expression of CD25 expression was determined by Western blot analysis. β-actin was used as a loading control. NT, not treated.

T-cells require two extracellular signals for activation; the first signal is delivered by the TCR and the second signal by co-stimulatory receptors. α4β1 is a known co-stimulatory receptor for T-cell activation [29–32]. Given that α4β1 outside-in signalling activated many of the same TCR ESC proteins associated with TCR signalling, these proteins may be involved in α4β1-mediated co-stimulation. Therefore we determined the involvement of TCR ESC proteins in α4β1-driven co-stimulation of the late T-cell activation marker CD25 was determined in Jurkat and naïve CD4^+^ T-cells. TCR stimulation alone did not induce CD25 expression in Jurkat and naïve CD4^+^ T-cells (Figures 7A and 7B). However, CD25 expression was induced by α4β1 outside-in signalling and was not further increased by α4β1 and TCR co-stimulation (Figures 7A and 7B). Therefore α4β1 provides a unique outside-in signal to induce CD25 expression in Jurkat and primary CD4^+^ T-cells.

We determined the involvement of TCR ESC proteins in α4β1-mediated CD25 response using TCR ESC knockout and reconstituted Jurkat cell lines. CD25 expression was not induced in JCAM1.6, P116 and J.Vav1 Jurkat cells stimulated by α4β1 outside-in signalling, but was restored in their respective reconstituted cell lines (Figure 7C). In contrast, α4β1-mediated CD25 expression was comparable between parental, J14 and JCAM2.5 Jurkat cells (Figure 7C). Therefore CD25 expression was dependent on Lck, ZAP-70 and Vav1, but not SLP-76 and LAT. We also determined the involvement of TCR ESC proteins in α4β1-mediated induction of CD25 in naïve CD4^+^ T-cells using Lck and ZAP-70 inhibitors (Lck inhibitor II and piceatannol respectively). Inhibition of either Lck or ZAP-70 suppressed α4β1-mediated CD25 expression in CD4^+^ T-cells (Figures 7D and 7E). α4β1 did not provide a co-stimulus for CD25, but rather delivered a distinct signal associated with T-cell activation that was dependent on TCR ESC proteins in both Jurkat and primary CD4^+^ T-cells.

### α4β1-mediated co-stimulation of CD69 requires TCR ESC proteins

**Figure 8:**
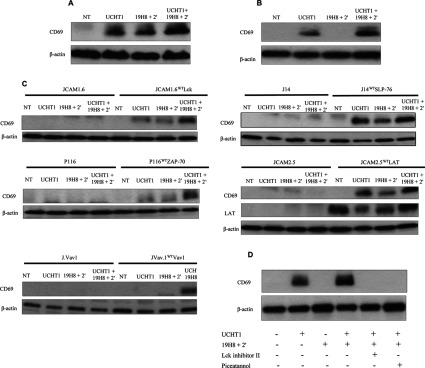
α4β1 outside-in signalling requires TCR ESC proteins to co-stimulate CD69 expression (**A**) Jurkat cells and (**B**) naïve CD4^+^ T-cells were stimulated with the anti-CD3 antibody UCHT1, the anti-α4β1 antibody 19H8 plus secondary cross-linker (2′) or UCHT1 in combination with 19H8 plus 2′ for 30 min. CD69 expression was determined by Western blot analysis. β-actin was used as a loading control. (**C**) Parental, Lck-deficient JCAM1.6, Lck-reconstituted JCAM1.6 (JCAM1.6^WT^Lck), ZAP-70-deficient P116, ZAP-70-reconstituted P116 (P116^WT^ZAP-70), Vav1-deficient J.Vav1, Vav1-reconstituted J.Vav1 (J.Vav1^WT^Vav1), SLP-76-deficient J14, SLP-76-reconstituted J14 (J14^WT^SLP-76), LAT-deficient JCAM2.5 and LAT-reconstituted JCAM2.5 (JCAM2.5^WT^LAT) Jurkat cells were stimulated with UCHT1, 19H8 plus 2′ or UCHT1 in combination with 19H8 plus 2′ for 30 min. Expression of CD69 expression was determined by Western blot analysis. β-actin was used as a loading control. (**D**) Naïve CD4^+^ T-cells were stimulated with UCHT1, 19H8 plus 2′, or UCHT1 in combination with 19H8 plus 2′ for 24 h. Cells were also pre-treated for 2 h with Lck inhibitor II (20 nM) or the ZAP-70 inhibitor piceatannol (50 μg/ml) prior to co-stimulation with UCHT1 and 19H8 for 24 h. Expression of CD69 expression was determined by Western blotting. β-actin was used as a loading control. NT, not treated.

In addition to CD25, we examined the effect of α4β1 stimulation and α4β1 co-stimulation on the expression of the early T-cell activation marker CD69. In Jurkat cells, CD69 expression was induced by α4β1 alone and UCHT1 alone (Figure 8A), whereas CD69 expression was not induced by α4β1 alone and slightly induced by TCR stimulation alone in CD4^+^ T-cells (Figure 8B). However, exposure to both α4β1 and TCR stimulation markedly increased CD69 expression compared with stimulation of either pathway alone in Jurkat and naïve CD4^+^ T-cells (Figures 8A and 8B). α4β1-mediated co-stimulation of CD69 expression was absent in JCAM1.6, P116, J.Vav1, J14 and JCAM2.5 cells, but was restored in their respective reconstituted cell lines (Figure 8C). Inhibition of either Lck or ZAP-70 blocked α4β1 co-stimulation of TCR-mediated CD69 expression in CD4^+^ T-cells (Figure 8D). Thus TCR ESC proteins are required for α4β1-mediated co-stimulatory signalling to CD69 in Jurkat and naïve CD4^+^ T-cells.

### α4β1-mediated CD25 and CD69 responses are ERK-independent

**Figure 9:**
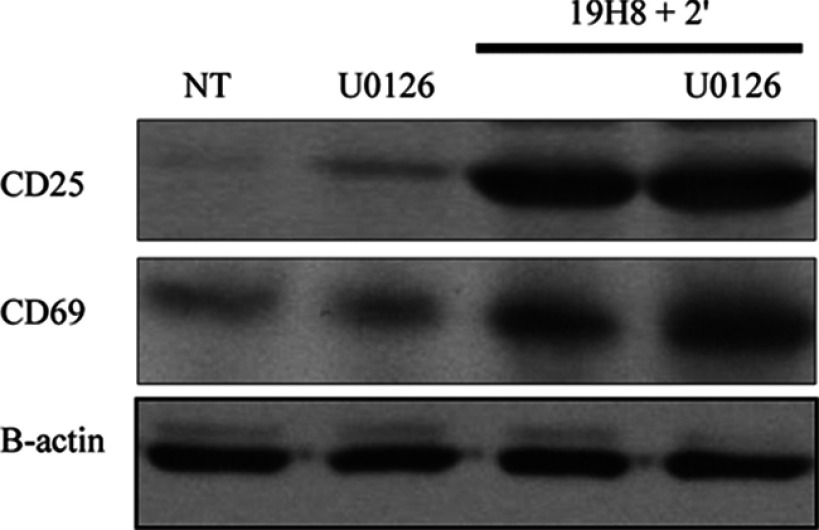
α4β1-mediated CD25 and CD69 signalling occur independently of ERK Jurkat cells were stimulated with the anti-α4β1 antibody 19H8 plus secondary cross-linker (2′) for 30 min. Jurkat cells were also pre-treated with U0126 (10 μM) for 2 h prior to the addition of 2′ for 30 min. CD25 and CD69 expression were determined by Western blot. β-actin was used as a loading control. NT (not treated) cells and cells treated with U0126 alone served as controls.

The MAPK ERK pathway has been linked to CD25 and CD69 expression in T-cells [33–36]. Therefore we determined whether ERK was involved in α4β1-mediated CD25 and CD69 signalling. Inhibition of ERK using U0126 had no effect on CD25 or CD69 expression induced by α4β1 outside-in signalling (Figure 9). Therefore the MAPK ERK pathway is not involved in α4β1-mediated CD25 or CD69 expression.

## DISCUSSION

Signal transduction in human cells is based on complex networks and feedback loops of protein interactions. Despite widespread literature on signal transduction from individual cell membrane receptors, little is known about the cross-talk between receptor pathways. TCR ESC proteins, initially thought to be exclusive to TCR signalling, have been shown to transduce signals from diverse receptors. Both IFNαR and CXCR4 cross-talk with the TCR through the utilization of a common subset of proteins that comprise the TCR ESC [4–7]. The results of the present study demonstrated that α4β1 outside-in and TCR signalling pathways are also networked through the use of TCR ESC proteins. However, the present study revealed key differences in the activation of TCR ESC proteins between α4β1 outside-in and TCR signalling pathways. In TCR signalling, activated Lck recruits ZAP-70 to the TCR, resulting in ZAP-70 autophosphorylation at Tyr^319^, binding to Lck, and subsequent phosphorylation of ZAP-70 at Tyr^493^ [13,14]. We found that Lck-dependent ZAP-70 autophosphorylation at Tyr^319^ was induced by α4β1 outside-in signalling. Lck-dependent phosphorylation of ZAP-70 at Tyr^319^ was also observed in LFA-1-mediated outside-in signalling in T-cells [37]. However, in contrast with TCR signalling, we found that ZAP-70 kinase function was not active in α4β1 outside-in signalling, as shown by the absence of ZAP-70 phosphorylation at Tyr^493^. Our results demonstrated that ZAP-70 mediates integrin outside-in signalling in T-cells by a mechanism distinct from its role in signalling downstream of the TCR. Furthermore, ZAP-70 kinase activity is critical in the transduction of signals from the TCR, but not from α4β1.

Activation of Vav1, SLP-76 and LAT is dependent on ZAP-70 in TCR signalling; however, this was not the case with α4β1 outside-in signalling. α4β1-mediated Vav1 and LAT phosphorylation were unaffected in ZAP-70-deficient P116 cells. Vav1 has been reported to interact with ZAP-70 in TCR signalling [38]. However, other studies have found that ZAP-70 does not directly interact with Vav1, but rather regulates the phosphorylation and interaction of SLP-76 with the Vav1 SH2 domain [21,39]. Vav1 was selectively recruited to SLP-76 microclusters and did not co-localize with ZAP-70 in TCR microclusters [39]. ZAP-70 phosphorylates SLP-76 at specific tyrosine sites (Tyr^113^ and Tyr^128^) that allow Vav1 SH2 domain binding and facilitate Vav1 recruitment to the TCR ESC for phosphorylation [15,21]. SLP-76 mediates integrin outside-in signalling in platelets in a similar manner to TCR signalling. SLP-76 was phosphorylated at Tyr^112^ and Tyr^128^ in response to αIIbβ3 outside-in signalling in platelets [40]. Furthermore, replacement of SLP-76 Tyr^112^/Tyr^128^ with phenylalanine inhibited Vav1 phosphorylation induced by αIIbβ3 outside-in signalling. Defective Vav1 phosphorylation has also been found in SLP-76-deficient neutrophils [41]. In the present study, Vav1 phosphorylation by α4β1 outside-in signalling in T-cells occurred independently of SLP-76 despite phosphorylation of SLP-76 at Tyr^113^ and Tyr^128^. However, our data do not exclude the possibility that SLP-76 may be involved in the recruitment and activation of Vav1 downstream of α4β1 signalling. SLP-76 has been implicated in LFA-1-mediated outside-in signalling; however, T-cells were stimulated with the CXCR4 ligand stromal cell-derived factor-1α to initiate outside-in integrin signals [42]. Therefore discriminating between the effects of LFA-1 outside-in signalling and CXCR4-mediated inside-out signalling was not possible. It is clear from our results that TCR ESC proteins display functionally distinct roles in TCR and α4β1 outside-in signalling pathways. The mechanism by which ZAP-70, Vav1, LAT and SLP-76 mediate integrin outside-in signalling in T-cells is distinct from that of TCR signalling. This selective assembly of signalling complexes may help cells to distinguish different stimuli and may explain the differentiation of the specific downstream responses of these pathways. Further investigation of the interactions of TCR ESC proteins and their recruitment to integrin-initiated signalling complexes is warranted.

As with TCR signalling, ERK activity was induced by integrin α4β1 outside-in signalling. Others have also reported that integrin outside-in signalling induced by antibody cross-linking and ECM binding activates ERK in cancerous and normal cells [22–24,43–45]. Assembly of the TCR ESC in response to TCR stimulation results in the activation of the MAPK ERK via the canonical Ras/Raf-1/MEK pathway. Integrin outside-in signalling has also been shown to stimulate Ras/Raf-1/MEK signalling to ERK [22–24]. However, we found that α4β1 outside-in signalling induced ERK activation via a Rap1/B-Raf/MEK pathway in Jurkat T-cells. B-Raf has been shown to be a key MEK/ERK activator in various cell types and to regulate integrin outside-in-mediated ERK activation through MEK [25,26,28,46,47]. B-Raf expression in T-cells is controversial owing to the conflicting reports of the activity and expression of this protein [48,49]. We found B-Raf to be highly expressed in Jurkat cells. Activation of B-Raf and ERK by the small G-protein Rap1 has been demonstrated in various cell types [27]. Rap1-mediated activation of ERK was demonstrated in B-Raf-transfected T-cells [50]. These different mechanisms of integrin outside-in ERK activation may reflect cell type-specific effects. Also, integrin-specific effects play a role in dictating the pathway of ERK activation in integrin outside-in signalling. Most of the studies examining integrin outside-in-mediated ERK activation have used the ECM substrates fibronectin and collagen. Integrins α4β1 and α5β1 can bind to fibronectin, and α2β1 can bind to collagen. Therefore the integrins that were activated in the previous studies may have been different from those in the present study. Furthermore, it appears that β1-integrins are connected to differential signalling pathways in T-cells and may regulate distinct T-cell functions. Despite a common β1 subunit, α2β1 and α4β1 were differentially recruited to lipid rafts in Jurkat cells [51]. Differential recruitment of these integrins may affect their signalling pathways by promoting or inhibiting the assembly of various downstream effectors.

We and others have previously demonstrated that IFNαR and TCR are networked such that IFNαR requires a functional TCR as well as Lck, ZAP-70, Vav1 and SLP-76 to induce ERK activation in Jurkat and primary CD4^+^ T-cells [1,2,5,7]. This has also been demonstrated for CXCR4-mediated ERK activation in Jurkat cells [6]. In the present study, we found that ERK activation induced by α4β1 outside-in signalling occurred independently of the TCR and TCR ESC proteins ZAP-70, SLP-76, Vav1 and LAT. However, the results from the present study suggest the involvement of Lck in α4β1 outside-in-mediated ERK activation. LFA-1-mediated ERK activation was also shown to be partially dependent on Lck in CD8^+^ T-cells [52]. In the present study, the effect of Lck on ERK activation was not due to different levels of α4β1 expression in Jurkat and JCAM1.6 cells, as JCAM1.6 cells reconstituted with Lck displayed similar ERK activity to that of parental Jurkat cells. Comparable levels of α4 and β1 subunits of the VLA-4 heterodimer were present in Jurkat and JCAM1.6 cells; however, JCAM1.6 cells exhibited defective adhesion to the VLA-4 ligands VCAM-1 (vascular cell adhesion molecule-1) and fibronectin [53].

Although the role of α4β1 as a T-cell co-stimulatory receptor has been established, the mechanism by which it exerts its co-stimulatory actions is less clear. Stimulation of α4β1 outside-in signalling with 19H8 and natural ligands (VCAM-1 and fibronectin) has been shown to stimulate TCR-dependent up-regulation of the T-cell activation markers CD69 and CD25 [54]. In the present study, we found that 19H8 co-stimulated TCR-mediated CD69 expression and this was dependent on TCR ESC proteins Lck, ZAP-70, Vav1, SLP-76 and LAT. Considering that integrins provide co-stimulatory signals to T-cells, the combination of TCR and α4β1 signalling networks with overlapping ESC components may serve to amplify minimal signals. SLP-76 was shown to be required for α4β1-dependent T-cell co-stimulation in Jurkat and primary T-cells [32,55]. T-cell co-stimulation by α4β1 prolonged the interaction of SLP-76 with ZAP-70 and increased the persistence of SLP-76 microclusters. These interactions could facilitate T-cell co-stimulation by preventing the inactivation of TCR-mediated SLP-76 microclusters and prolonging the transmission of TCR-mediated signals. In contrast with previous reports, we found that α4β1 did not provide a co-stimulus for CD25, but rather delivered a distinct signal associated with T-cell activation in both Jurkat and primary CD4^+^ T-cells. As with CD69 co-stimulation, the induction of CD25 expression by α4β1 outside-in signalling was dependent on TCR ESC proteins. T-cell activation or differentiation state has been shown to differentially affect co-stimulation requirements. Differences in the response of memory and naïve T-cells to co-stimulatory molecules including integrins (β1, β2 and β7) and CD28 have been reported [56,57]. In addition, CD4^+^ and CD8^+^ T-cell subsets differed in their co-stimulatory responses to CD28 and intercellular adhesion molecule-1 [58,59]. Therefore discrepancies in α4β1 co-stimulation of CD25 expression between the present study and previous reports may reflect T-cell subset-specific differences in integrin function and co-stimulation.

**Figure 10:**
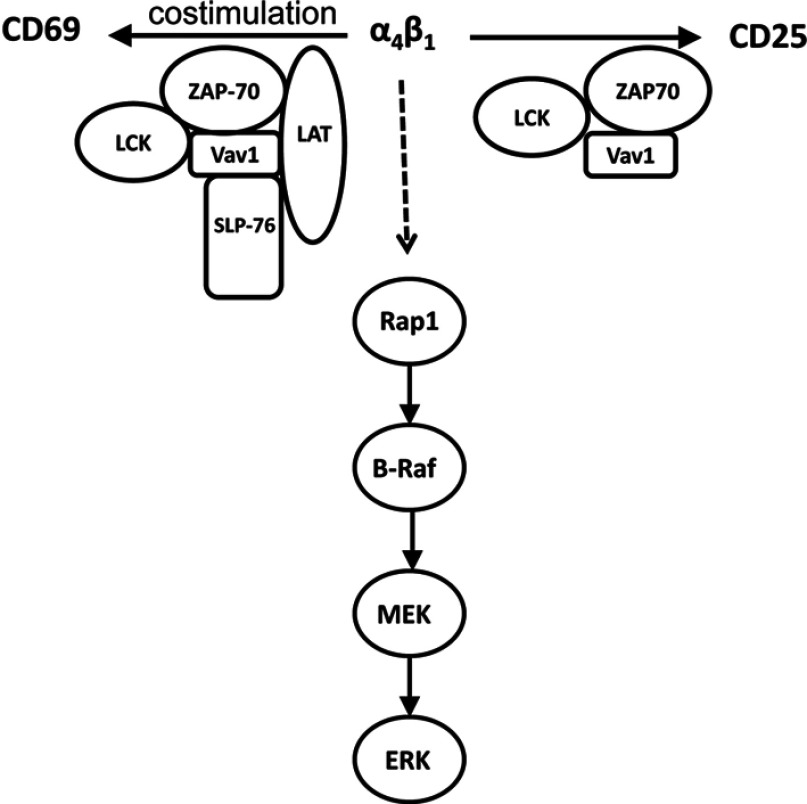
Schematic representation of the differential requirement of TCR ESC proteins in α4β1 outside-in signalling in T-cells α4β1 outside-in signalling induced CD25 expression, co-stimulation of CD69 expression and activation of the MAPK ERK pathway. Unlike TCR signalling, ERK activation occurred independently of TCR ESC proteins. In contrast, TCR ESC proteins were required for the induction of CD25 and co-stimulation of CD69 by α4β1 outside-in signalling. Lck, ZAP-70 and Vav1 were required for CD25 induction and CD69 co-stimulation by α4β1 outside-in signalling; however, SLP-76 and LAT were only required for CD69 co-stimulation.

In summary, α4β1 outside-in signalling induced several signalling events in T-cells, including activation of the MAPK ERK pathway and pathways leading to the induction of CD69 and CD25 (Figure 10). CD25 induction and CD69 co-stimulation by α4β1-outside in signalling were not mediated by ERK. Therefore CD25, CD69 and MAPK ERK represented three distinct pathways activated by α4β1 outside-in signalling. These pathways displayed a differential requirement for TCR ESC proteins. The MAPK ERK pathway of Rap1/B-Raf/MEK induced by α4β1 outside-in signalling occurred independently of TCR ESC proteins. However, TCR ESC proteins were required for CD25 induction and CD69 co-stimulation by α4β1 outside-in signalling. Interestingly, TCR ESC proteins had overlapping and distinct functions in integrin-mediated CD25 and CD69 signalling pathways. Lck, ZAP-70 and Vav1 were involved in both α4β1-mediated CD25 induction and CD69 co-stimulation. On the other hand, SLP-76 and LAT were only involved in α4β1-mediated CD69 co-stimulation. The present study provides new insights into integrin outside-in signalling in T-cells and the cross-talk of this with TCR signalling. Aberrancies in TCR and integrin signalling pathways are associated with immunodeficiency, cancer and autoimmune diseases. Understanding the molecular basis underlying the communication between these signalling networks will be valuable in developing targeted therapies for integrin and T-cell-based disorders.
